# The Clinical Utility of Next-Generation Sequencing in Childhood and Adolescent/Young Adult Solid Tumors: A Systematic Review and Meta-Analysis

**DOI:** 10.3390/cancers17081292

**Published:** 2025-04-11

**Authors:** Lior Katz, Myriam Ben-Arush, Einav Blanche, Inbar Meir, Oz Mordechai

**Affiliations:** 1 Pediatrics, Ruth Rappaport Children’s Hospital, Rambam Medical Center, Haifa 3109601, Israel; 2 Joan and Sanford Weill Pediatric Hematology Oncology and Bone Marrow Transplantation Division, Ruth Rappaport Children’s Hospital, Rambam Medical Center, Haifa 3109601, Israel

**Keywords:** NGS, precision medicine, solid tumors

## Abstract

Background: Next-generation sequencing (NGS) has emerged as a transformative tool in precision medicine, offering insights into actionable genomic alterations and informing clinical decision-making in childhood and adolescent/young adult (AYA) solid tumors. Methods: We conducted a systematic review and meta-analysis to assess the utility of NGS in identifying actionable genomic alterations and its impact on clinical decision-making. Studies involving patients aged 0–40 years with solid tumors were included. Data were extracted using Covidence, and pooled estimates were calculated using a random-effects model. Bias was assessed using Begg–Mazumdar, Egger, and Harbord tests. Results: Out of 13,624 references screened, 24 studies met eligibility criteria, comprising 5278 patients and 5359 samples, of which 5207 provided usable data. The pooled proportion of actionable alterations was 57.9% (95% CI: 49.0–66.5%), with minimal evidence of publication bias. Clinical decision-making outcomes were reported in 21 studies, with a pooled proportion of 22.8% (95% CI: 16.4–29.9%). Germline mutation rates, reported in 11 studies, yielded a pooled proportion of 11.2% (95% CI: 8.4–14.3%), consistent with rates typically observed in childhood cancers. Significant heterogeneity was observed across studies due to differences in sequencing methodologies, tumor types, and sampling strategies. Conclusions: NGS demonstrates considerable potential in identifying actionable genomic targets and guiding clinical decision-making in childhood and AYA solid tumors. However, the variability in methodologies underscores the need for standardized protocols and reporting practices to enhance comparability and generalizability. This meta-analysis highlights the promise of genomic medicine while acknowledging challenges posed by heterogeneity in study designs.

## Simple Summary

Childhood cancers, although rare, remain a leading cause of disease-related deaths in children and young adults. New genetic testing methods, such as next-generation sequencing (NGS), have emerged, allowing us to better understand the genetic makeup of these cancers. Our research was designed to evaluate the utility of these methods in identifying genetic changes that could potentially inform treatment decisions. By reviewing multiple studies involving young patients with solid tumors, we found that genetic testing frequently identified significant genetic alterations and informed treatment choices in many cases. However, variability among studies highlighted the need for consistent guidelines and methods in genetic testing and reporting. Our findings suggest that while genetic testing has substantial potential for improving care, future research should focus on standardizing these methods to maximize benefits for young patients with cancer and help clinicians make more effective treatment decisions.

## 1. Introduction

Pediatric cancers are uncommon, with an estimated annual incidence of approximately 189 new cases per million children aged 0–19 years. Although survival rates for childhood malignancies have improved significantly, cancer remains the leading cause of disease-related death among children beyond infancy [1]. Prognosis remains particularly poor for specific tumor groups, notably, solid malignancies such as high-grade glioma, brainstem tumors, high-risk medulloblastoma, metastatic sarcomas, and high-risk neuroblastoma. Outcomes further worsen with relapse, given the lack of standardized guidelines for refractory disease management [2,3,4,5].

Precision medicine approaches aim to treat cancer by targeting specific genetic aberrations identified within individual cancer cells. Genomic profiling allows clinicians to detect oncogenic drivers and pathogenic variants, providing personalized therapeutic strategies [6]. Despite their infancy, precision medicine methods hold great promise in pediatric oncology, fostering optimism toward predictive biomarker-driven trials and more reliable prognosis determination [7].

## 2. Pediatric Solid Tumors and Actionable Mutations

Pediatric solid tumors constitute a diverse group, prominently including central nervous system tumors, sarcomas, bone and kidney tumors, peripheral nervous system tumors, and germ cell tumors [8,9,10,11]. Compared with adult malignancies, pediatric tumors often originate from embryonic tissues, displaying distinctive genomic profiles characterized by relatively low mutational burdens and fewer recurrent mutations [12,13,14,15]. This unique genomic landscape poses both opportunities and challenges in precision oncology his distinct genomic landscape creates both opportunities and limitations for precision oncology—on one hand, by exposing molecular vulnerabilities that may be therapeutically exploited, and on the other, by restricting the number of actionable targets available for intervention.

Actionable mutations, defined as genomic alterations that are potentially responsive to targeted therapies, differ significantly from driver mutations, which confer selective growth advantages without indicating current therapeutic targets [16,17,18]. Typical somatic alterations in pediatric tumors involve signaling pathways such as RTK (*EGFR*), MAPK (*KRAS*), and PI3K-mTOR (*PTEN*), transcriptional regulators like *MYC/MYCN*, DNA repair genes (*TP53*), and epigenetic modifiers (ATRX) [6]. Germline pathogenic variants primarily involve genes like *TP53, BRCA1/2, NF1, RB1, WT1*, and *APC*, among others. These variants are distributed across multiple pediatric tumor types, including sarcomas, brain tumors, neuroblastoma, and Wilms tumors, highlighting the critical role of germline genetics in pediatric oncology.

Due to the inherent rarity of childhood solid tumors, along with the lower frequency and distinct nature of their genomic alterations, and the fact that they usually require reduced drug dosages, early clinical trials lag behind those of adult cancers in terms of practical and financial factors [17,18].

Another important aspect of pediatric cancer in general concerns treatment-related comorbidities. Nearly two-thirds of long-term survivors continue to experience treatment-related late effects and toxicities, including neurocognitive deficits, obesity, premature aging, organ toxicity, and secondary neoplasms, among others [19]. Consequently, there has been a paradigm shift in the approach to pediatric oncology in the last several years, with two primary objectives. The first is to continue to improve cure rates in pediatric cancers, particularly for subtypes that have a poor prognosis despite multimodal therapy. The second is to maintain cure rates while simultaneously reducing the toxic burden of therapy in children and adolescents, thereby providing them with better long-term outcomes with minimal late effects. The application of precision medicine principles to the practice of pediatric oncology provides an excellent approach to achieving these two goals [20].

### 2.1. Clinical Utility of Precision Oncology in Pediatric Tumors

Globally, most children initially receive intensive chemotherapy and may receive radiation as the standard of care; relapsed tumors often harbor additional mutations, including druggable targets [21]. However, pediatric cancer genomes at relapse remain insufficiently studied, partly due to limited biopsy availability and ethical concerns surrounding invasive procedures in children. Moreover, ethical implications arise from incidental germline findings during genomic profiling, necessitating careful consideration and genetic counseling protocols [22].

Clinical experiences with targeted therapies in pediatric solid tumors have demonstrated promising, though variable, outcomes. Mutations in genes such as *BRAF, ALK, EGFR, FGFR*, and *NTRK* fusions have shown potential responsiveness to targeted agents. However, current clinical experience remains limited, and clinical trials evaluating response rates, progression-free survival (PFS), and overall survival (OS) are ongoing, emphasizing the importance of continued research.

### 2.2. Need for Standardized Protocols

A significant challenge identified in this systematic review is the substantial variability observed among the included studies regarding methodological approaches, which markedly influences the interpretation and comparability of results. Variability arose from multiple aspects, including differences in sequencing techniques such as targeted NGS panels, whole-exome sequencing (WES), whole-genome sequencing (WGS), RNA sequencing, methylation profiling, and fluorescence in situ hybridization (FISH). Each of these genomic profiling methods has distinct analytical sensitivities, specificities, and capabilities, resulting in differing abilities to detect clinically relevant genomic alterations. Furthermore, significant differences existed in tumor sampling strategies, with some studies analyzing samples exclusively from primary diagnoses and others focusing specifically on relapsed or refractory diseases. Such differences in sample selection not only influence the genomic profiles obtained but also impact the proportion of actionable findings reported, as tumors at relapse often harbor different mutation landscapes compared to primary diagnoses.

Additionally, heterogeneity in reporting standards and definitions of “actionable alterations” further complicates direct comparison across studies. The lack of consistent criteria for clinical actionability results in a broad spectrum of interpretations, making pooled analyses challenging and potentially reducing clinical relevance. To mitigate these issues, future research should strongly emphasize the standardization of sequencing methodologies, sample collection practices, and the establishment of consistent, clinically meaningful reporting standards. Relevant existing guidelines developed by international oncology organizations, such as the European Society for Medical Oncology (ESMO), American Society of Clinical Oncology (ASCO), and Children’s Oncology Group (COG), provide valuable structured frameworks that can significantly enhance methodological consistency. Additionally, implementing standardized classification systems for defining clinical actionability—such as the ESMO Scale for Clinical Actionability of Molecular Targets (ESCAT) or the OncoKB knowledgebase—can further harmonize the interpretation of genomic results, facilitating more evident comparisons across different studies and promoting broader clinical implementation. These standardization efforts will ultimately enable more robust meta-analyses, improve clinical decision-making, and enhance the translational impact of precision oncology research in pediatric cancer.

## 3. Methods

### 3.1. Study Design and Search Strategy

This study followed a systematic review and meta-analysis approach to evaluating the yield of next-generation sequencing (NGS) testing in childhood and adolescent/young adult (AYA) solid tumors. A systematic literature search was conducted across multiple databases, including [PubMed, Google Scholar, and citation searching], to identify relevant studies published until December 2024. The search strategy included combinations of terms such as “next-generation sequencing”, “genomics”, “precision medicine”, “targeted therapy”, “childhood cancer”, and “AYA solid tumors”, tailored to each database. The Preferred Reporting Items for Systematic Reviews and Meta-Analyses (PRISMA) guidelines were followed throughout the review process.

### 3.2. Eligibility Criteria

Studies were eligible for inclusion if they met the following criteria:

- Population: Pediatric and AYA patients (aged 0–40 years) diagnosed with solid tumors.
- Intervention: Next-generation sequencing (NGS) as part of genomic testing, precision medicine, or targeted therapy approaches.
- Outcome: Reported proportions of actionable mutations and/or decision-making based on NGS findings.
- Study Design: Original studies, including observational studies, clinical trials, and retrospective cohorts.

### 3.3. Screening and Data Extraction

The systematic review process was conducted using Covidence software to streamline study screening and data extraction [23]. Two authors independently screened titles and abstracts, followed by full-text assessments to determine eligibility. Discrepancies were resolved through consensus. Extracted data included the following:

- Study characteristics: Author, year, journal, trial name, study methods, location, and age.
- NGS metrics: Number of patients, samples, actionable mutation rates, decision-making rates, and germline mutation rates (when reported).

Data relevant to solid tumors were extracted for studies involving both solid and non-solid tumors (e.g., combined trials). The exclusion criteria included studies focusing exclusively on hematological malignancies, non-solid tumor cohorts, animal models, or review articles without original data.

### 3.4. Data Analysis

Data synthesis was conducted using a random-effects model (DerSimonian-Laird) to account for heterogeneity among studies. The primary outcomes included the pooled proportion of actionable genomic alterations and decision-making outcomes based on next-generation sequencing (NGS) findings. Heterogeneity was assessed using the I^2^ statistic and Cochran’s Q test. Funnel plots and the Harbord test were used to assess publication bias.

Publication bias was evaluated using Funnel plots, the Harbord test, Begg–Mazumdar rank correlation, and Egger’s regression asymmetry test. All statistical analyses were performed using StatsDirect software (Version 4) [24], with significance levels set at *p* < 0.05.

We used the PRISMA checklist when writing our report [25].

### 3.5. Ethical Considerations

This study is based on a review of published data; hence, ethical approval was not required.

## 4. Results

### Study Selection

The systematic review process initially identified a total of 13,624 references through comprehensive searches of electronic databases (including PubMed and Google Scholar) and citation searching, using predefined search terms and criteria. Following this initial step, 3326 duplicate records were removed, leaving 10,297 unique references for subsequent screening. These references were independently screened based on their titles and abstracts by two authors using Covidence software [23]. This process aimed to exclude studies clearly outside of the scope of this meta-analysis, such as those involving exclusively hematological malignancies or non-human models or lacking original data. Following the title and abstract screening, 75 studies were identified as potentially relevant and selected for detailed full-text evaluation. During the full-text review, each study was rigorously assessed for eligibility against predefined inclusion criteria, specifically targeting pediatric and adolescent/young adult (AYA) populations (ages 0–40 years) with solid tumors who underwent genomic profiling using next-generation sequencing (NGS). Studies had to clearly report relevant outcomes, such as the proportion of actionable genomic alterations or the proportion of cases influencing clinical decision-making. Studies failing to meet these criteria, such as those lacking explicitly reported outcome data or exclusively involving non-solid tumors, were excluded at this stage.

**Figure 1:**
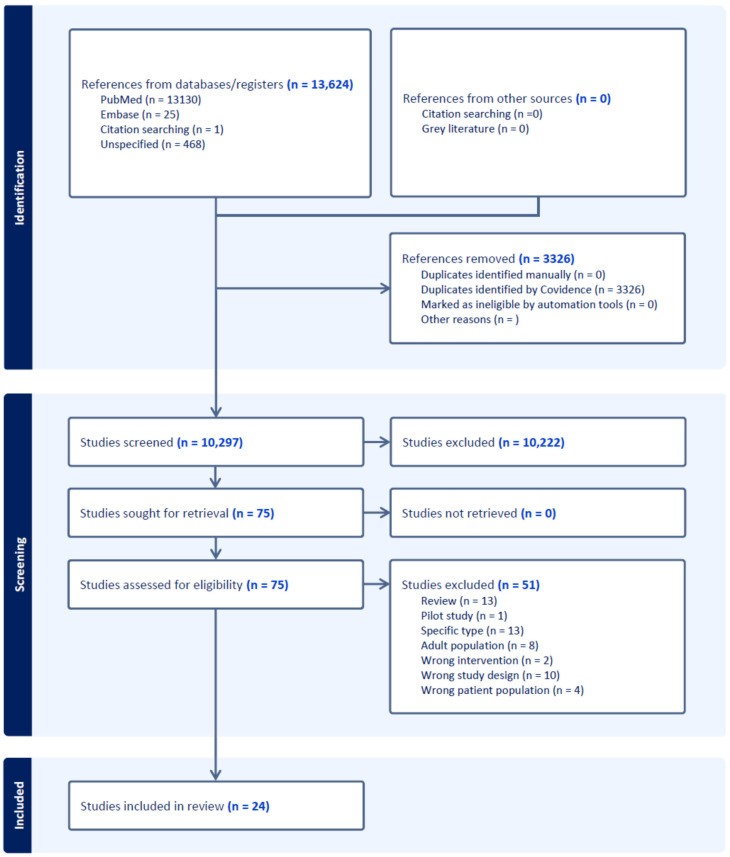
Study selection process following PRISMA guidelines. It includes the number of records identified, duplicates removed, and studies screened. After assessing 75 full-text articles for eligibility, 24 studies were included in the meta-analysis.

Ultimately, after careful review and consensus discussions between authors to resolve any discrepancies, 24 studies were found to fully meet all inclusion criteria and were therefore included in the final meta-analysis. A comprehensive and detailed list of all included studies, summarizing their key characteristics, is provided in Appendix A to enhance transparency and reproducibility. The complete process of study identification, screening, assessment for eligibility, and inclusion in the final meta-analysis is clearly illustrated in the PRISMA flow diagram (Figure 1). This diagram visually summarizes each step of the systematic review, explicitly showing the reasons for exclusion at each stage, thus facilitating a transparent and reproducible review process.

A total of 5278 patients were included across the 24 studies analyzed, contributing 5359 samples. Among these, 5207 samples successfully yielded data for analysis. The median of the reported median ages across studies was 11.5 years.

**Figure 2:**
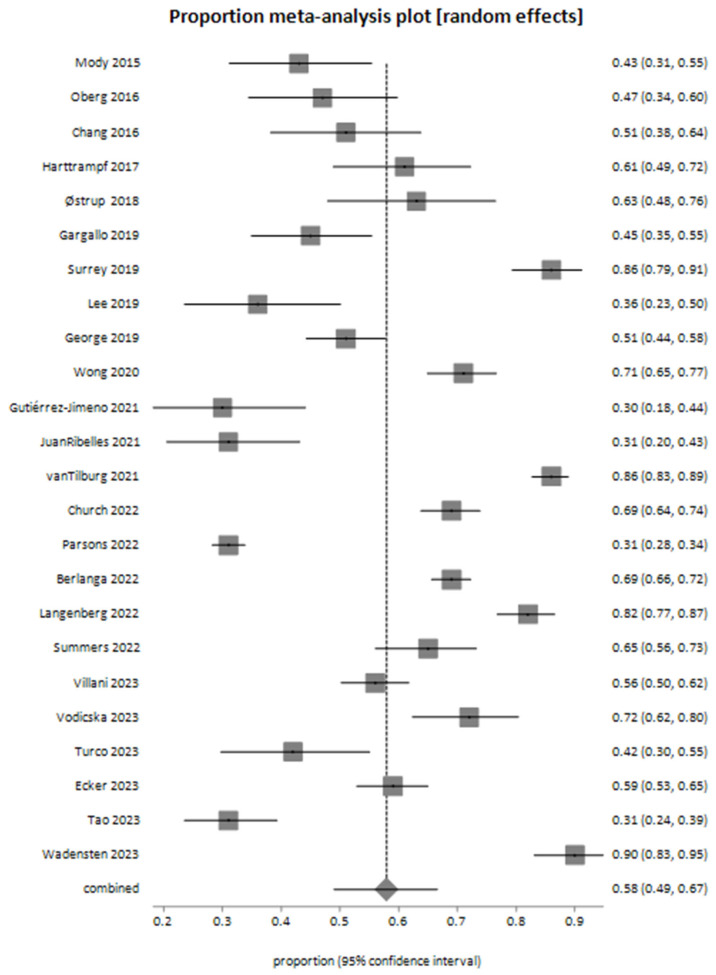
Meta-analysis plot illustrating the pooled proportion of actionable genomic alterations identified through next-generation sequencing (NGS) across the included studies [26,27,28,29,30,31,32,33,34,35,36,37,38,39,40,41,42,43,44,45,46,47,48,49].

The meta-analysis for actionable genomic alterations yielded a pooled proportion of 0.578982 (95% CI: 0.490044 to 0.665408) using the random-effects model (Figure 2).

**Figure 3:**
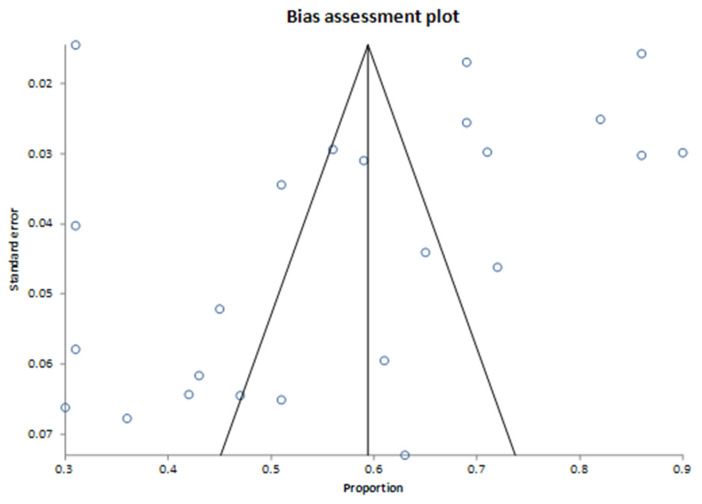
Bias assessment plot for findings on actionable genomic alterations. The pooled proportion of actionable findings derived from 24 studies.

The assessment of publication bias is presented in the bias assessment plot (Figure 3). The tunnel plot appeared symmetric, suggesting limited evidence of publication bias. Bias assessment indicated minimal evidence of publication bias, with the Harbord test showing no significant bias, with a bias estimate of 0.222842 (92.5% CI: −5.127396 to 5.573079, *p* = 0.9387). The Begg–Mazumdar test detected a weak correlation (Kendall’s tau = −0.326087, *p* = 0.0228), indicating potential publication bias. The Egger test did not suggest significant bias, with a bias estimate of −2.105101 (95% CI: −8.408872 to 4.198669, *p* = 0.4958). The four studies observed close to the *Y*-axis (with proportions around 0.3 to 0.4 and high standard error, ~0.06–0.07) likely represent smaller studies with relatively lower proportions of actionable mutations. The four studies clustered in the upper right quadrant (proportions around 0.7 to 0.9 with low standard error ~0.02–0.04) represent studies with larger sample sizes and/or high proportions of actionable mutations. Their deviation from the central pooled estimate could indicate genuine differences in populations or methods (e.g., tumor type selection or sequencing technology) contributing to higher actionability rates rather than publication bias.

**Figure 4:**
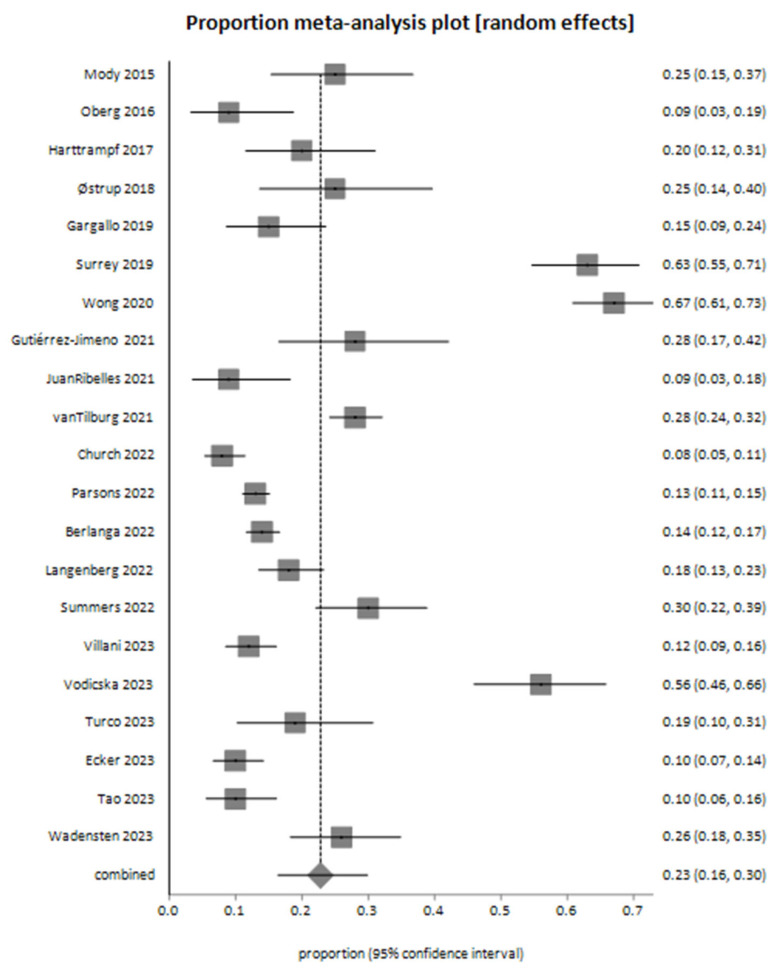
Meta-analysis plot for decision-making outcomes based on next-generation sequencing (NGS) findings—the pooled proportion of decision-making events derived from 21 studies [26,28,29,30,31,34,35,36,37,38,39,40,41,42,43,44,45,46,47,48,49].

The pooled proportion of decision-making events based on next-generation sequencing (NGS) results, estimated using a random-effects model (DerSimonian-Laird), was 0.228102 (95% CI: 0.164242 to 0.299019). These results were derived from 21 studies, as illustrated in the meta-analysis plot (Figure 4).

While some tests (e.g., Egger and Begg–Mazumdar) suggested possible publication bias for decision-making, the Harbord test showed no significant evidence of bias: The Harbord test showed no statistically significant bias, with a bias estimate of 3.685992 (92.5% CI: −2.007909 to 9.379893, *p* = 0.2376). The Begg–Mazumdar test suggested moderate evidence of publication bias with Kendall’s tau = 0.419048 (*p* = 0.0074). The Egger test indicated potential bias with a bias estimate of 4.852385 (95% CI: 0.421871 to 9.282899, *p* = 0.0335).

**Figure 5:**
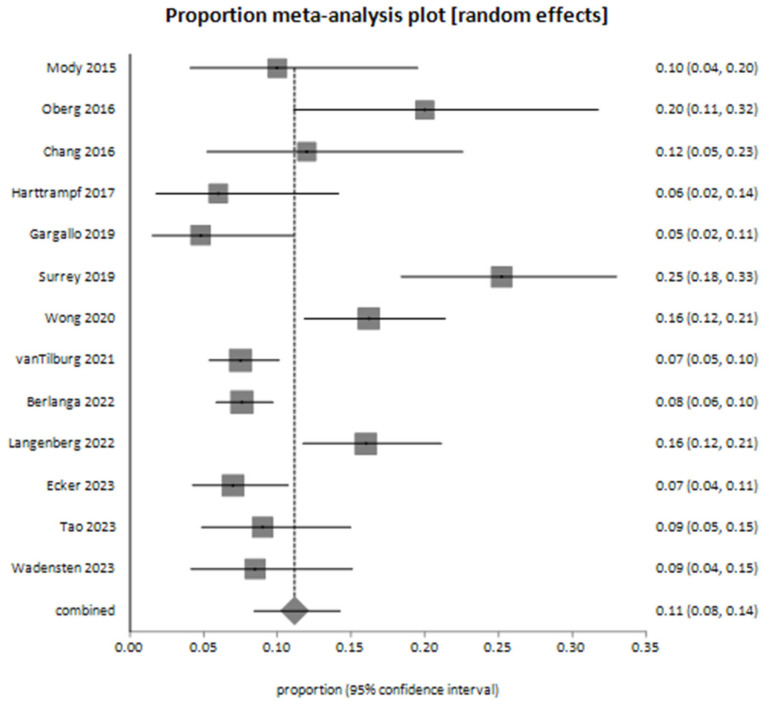
Meta-analysis plot for suspected germline mutations among patients—the pooled proportion of decision-making events derived from 13 studies [26,27,28,29,31,34,35,38,39,41,44,45,49].

The meta-analysis for suspected germline mutations, based on 13 studies, yielded a pooled proportion of 0.111697 (95% CI: 0.084192 to 0.142541) using the random-effects model (Figure 5). Bias assessment indicated no firm evidence of significant publication bias. The Harbord test showed *p* = 0.2781, the Begg–Mazumdar test showed *p* = 0.0573, and the Egger test showed *p* = 0.0502, approaching statistical significance.

## 5. Discussion

Our findings underscore the significant potential of next-generation sequencing (NGS) in identifying clinically actionable genomic targets in childhood and adolescent/young adult (AYA) solid tumors. The pooled proportion of actionable alterations was 57.9%, highlighting the substantial opportunities offered by genomic technologies to advance precision oncology in pediatric oncology. Furthermore, approximately 22.8% of cases directly benefited from NGS findings, which guided clinical decision-making and reinforced the clinical relevance and potential utility of incorporating genomic profiling into standard practice.

Despite these promising results, caution is warranted in interpretation due to the considerable heterogeneity identified across studies. The observed variability arose from differences in patient populations, tumor types, methodological approaches (including NGS panels, whole-exome sequencing [WES], whole-genome sequencing [WGS], RNA sequencing, methylation profiling, and FISH), and sample preparation methods. Differences in the timing of genomic assessments, notably between primary tumors and relapsed disease, further contributed to heterogeneity. Importantly, our findings reflect the inherent real-world diversity of practices, highlighting both the opportunities and challenges in achieving standardized, effective precision medicine strategies.

The definition of “actionable” genomic alterations itself varied considerably among the included studies. While our meta-analysis relied on authors’ criteria for actionability, this lack of standardization complicates direct comparisons. Implementing standardized definitions, such as those provided by OncoKB, ESCAT, or Pediatric MATCH, is essential to clearly delineate genuinely actionable alterations from variants lacking immediate clinical significance, thereby facilitating more reliable data synthesis and clearer clinical guidance.

Moreover, the clinical utility of NGS results may depend significantly on the infrastructure available for interpretation and clinical implementation. Centralized molecular tumor boards (MTBs), comprising multidisciplinary experts, likely enhance clinical utility by providing a uniform interpretation of genomic data, consistent treatment recommendations, and the systematic follow-up of clinical outcomes. Conversely, decentralized reporting may diminish the effectiveness of NGS findings in clinical decision-making due to variability in interpretation and implementation practices.

Another important consideration is the nature and quality of tissue samples analyzed. Fresh-frozen tissues typically yield superior-quality genomic material compared to formalin-fixed paraffin-embedded (FFPE) samples, particularly regarding RNA-based assays. Thus, standardization in sample preparation and handling protocols would further enhance the reliability and clinical applicability of genomic results. Future guidelines and research should explicitly address optimal sample preparation practices to improve data quality and clinical relevance.

Longitudinal or paired analyses of primary and relapse samples offer significant clinical value, enabling the identification of acquired mutations and resistance mechanisms not detectable at diagnosis. Such studies can highlight emerging therapeutic targets and mechanisms underlying tumor evolution and resistance, thereby providing essential insights into personalized treatment strategies. Unfortunately, pediatric tumor samples at relapse remain understudied, partly due to the limited feasibility of repeat biopsies, ethical considerations, and clinical hesitancy surrounding invasive procedures in children. However, these challenges also underline the importance of innovative, less invasive approaches such as liquid biopsies, which warrant future investigation.

Furthermore, while our pooled estimate of suspected germline mutations (11.2%) aligns well with previously published data [50], variability in reporting standards across studies remains a limitation. Ethical concerns regarding incidental germline findings not related to cancer predisposition further complicate genomic profiling, necessitating careful genetic counseling and ethical frameworks to handle these sensitive issues appropriately.

Additionally, the incremental benefit of NGS over standard diagnostic methods remains incompletely defined. Although NGS provides broader genomic coverage and can identify less anticipated yet potentially actionable mutations, standard diagnostic approaches such as IHC or FISH may adequately detect clinically significant alterations (e.g., *NTRK* fusions). Comparative studies assessing clinical outcomes between NGS and traditional methods are crucial in elucidating the actual incremental value of comprehensive genomic profiling.

Lastly, while our rigorous bias assessment employing multiple statistical methods (Begg–Mazumdar, Egger’s, and Harbord tests) demonstrated minimal publication bias, subtle biases associated with methodological differences remain possible. Clarifying the rationale behind using multiple tests ensures transparency and robustness in interpreting results.

Despite these limitations and methodological complexities, our systematic review and meta-analysis demonstrate the promising potential of NGS-based precision medicine for pediatric and AYA solid tumors. Continued efforts to harmonize methodologies, standardize reporting, and improve the clinical translation of genomic data will maximize the benefits of precision oncology and ultimately lead to better outcomes for young cancer patients.

## 6. Conclusions

Our systematic review and meta-analysis underscore the significant potential of next-generation sequencing (NGS) in identifying clinically actionable genomic alterations and informing treatment decisions in pediatric and adolescent/young adult (AYA) solid tumors. While our analysis revealed a substantial pooled proportion of actionable alterations (57.9%) and meaningful clinical impacts in approximately 22.8% of cases, the results should be interpreted cautiously due to notable methodological and clinical heterogeneity among studies.

The variability in definitions of “actionability”, patient populations, tumor types, genomic methodologies, the timing of sample collection, and tissue handling practices presents ongoing challenges for data synthesis and clinical implementation. Addressing these challenges will require the adoption of standardized reporting guidelines, consistent definitions of actionable genomic findings, and clear criteria for interpreting clinical relevance. Moreover, future research should prioritize prospective studies, longitudinal sampling, comparative effectiveness analyses (NGS versus traditional diagnostics), and careful consideration of ethical implications related to germline incidental findings.

By systematically addressing these critical issues, the pediatric oncology community can significantly enhance the clinical utility and translational impact of genomic profiling, ultimately improving outcomes and precision treatment strategies for young patients with cancer.

## Data Availability

All data included in this systematic review and meta-analysis were extracted from previously published articles. No new primary data were generated or analyzed. Details of included studies are provided in the manuscript. Additional data or clarifications can be requested from the corresponding author upon reasonable request.

## Appendix A. Summary of Studies Included in the Meta-Analysis

**Table A1:** Characteristics of included studies.

Study ID	Trial Name	Journal	Year	Age Range	Median Age	Countries	Methods
Mody 2015 [26]	Peds-MiOncoSeq	*JAMA*	2015	0–22	12	USA	WES; RNA
Chang 2016 [27]	POB of NCI	*Clin Cancer Res*	2016	0.7–25	15	USA	WES; WTS—whole-transcriptome sequencing; SNP Array
Oberg 2016 [28]	PIPseq	*Genome Medicine*	2016	0.2–26	8	USA	NGS panel; WES; RNA; WTS—whole-transcriptome sequencing
Harttrampf 2017 [29]	MOSCATO-01	*Clinical Cancer Research Online*	2017	0.8–24.3	10.9	France	NGS panel; WES; RNA
Østrup 2018 [30]		*Frontiers in Pediatrics*	2018	0–17	9.5	Other	WES; RNA; SNP Array
Gargallo 2019 [31]	Spanish initiative	*Translational Medicine Communications*	2019	0–25	9.9	Spain	NGS panel; SNP Array; IHC
George 2019 [32]	SMPAEDS	*European Journal of Cancer*	2019	0–24		United Kingdom	NGS panel
Lee 2019 [33]	SMC	*PLOS ONE*	2019	0.8–20.7	10.7	Other	NGS panel
Surrey 2019 [34]		*Genome Medicine*	2019	0–26	8.6	USA	NGS panel; RNA
Wong 2020 [35]	Zero Childhood Cancer Program	*Nature Medicine*	2020	0–31	10	Australia	WGS; WTS—whole-transcriptome sequencing; methylation array
Gutiérrez-Jimeno 2021 [36]		*Cancers*	2021	0–30.8	11.8	Spain	NGS panel
JuanRibelles 2021 [37]		*Journal of Personalized Medicine*	2021	1–18	11.5	Spain	NGS panel
vanTilburg 2021 [38]	INFORM	*Cancer Discovery*	2021	0–36	13	International	WES; WGS; methylation array
Berlanga 2022 [39]	MAPPYACTS	*Cancer Discovery*	2022	0.5–38.5	11.6	France; Spain; Ireland; Italy	WES; RNA
Church 2022 [40]	GAIN/iCAT2	*Nature Medicine*	2022	0–27.5	12	USA	NGS panel
Langenberg 2022 [41]	iTHER	*European Journal of Cancer*	2022	0.9–23	13.1	Germany	WES; RNA
Parsons 2022 [42]	Pediatric MATCH Trial	*Journal of Clinical Oncology*	2022	1–21	13	USA	NGS panel; RNA; IHC
Summers 2022 [43]	Aflac Precision Medicine Program (APMP)	*JCO Precision Oncology*	2022	0.2–25.7	12.1	USA	WES; RNA
Ecker 2023 [44]	The Pediatric Targeted Therapy 2.0 registry	*European Journal of Cancer*	2023	0–54	10	International	NGS panel; RNA; methylation array; IHC
Tao 2023 [45]	TOP-GEAR Project	*JCO Precision Oncology*	2023	1–28	12	Japan	NGS panel
Turco 2023 [46]		*Pediatric Blood & Cancer*	2023	0.9–21		USA	NGS panel; RNA
Villani 2023 [47]	SickKids Cancer Sequencing (KiCS) program	*Nature Cancer*	2023	0–38	7.1	Canada	NGS panel; RNA
Vodicska 2023 [48]		*World Journal of Pediatrics*	2023	0–21	8	Other	NGS panel; WES
Wadensten 2023 [49]		*JCO Precision Oncology*	2023	0–18		Other	WGS; RNA

**Table A2:** Outcomes of included studies: number of patients/samples, percentage of actionable alterations identified in each study, the proportion of cases where NGS findings impacted clinical decisions, and reported germline mutations in included studies.

Study ID	Number of Patients	Samples	Number of Sequencing Analysis Samples	% Failure	% Actionable Alterations	% Decision-Making Relevant to Test Results	% Suspected Germline
Mody 2015 [26]	70	70	63	10	42.8	25	10
Chang 2016 [27]	64	59	59	8	51		12
Oberg 2016 [28]	65	77	77		47	9.2	20
Harttrampf 2017 [29]	73	73	69		61	20.3	6
Østrup 2018 [30]	48	46	46		63	25	
Gargallo 2019 [31]	98	84	84	14.3	45	15	4.8
George 2019 [32]	223	255	209	18	51		
Lee 2019 [33]	55	55	55	3.6	36.4		
Surrey 2019 [34]	147	154	154		86.3	62.6	25.2
Wong 2020 [35]	247	252	252		71	67	16.2
Gutiérrez-Jimeno 2021 [36]	53	55	55		30	28	
JuanRibelles 2021 [37]	70	70	70		31	9	
vanTilburg 2021 [38]	519	519	519	6	85.9	28.3	7.5
Berlanga 2022 [39]	774	833	695	16	69	13.8	7.6
Church 2022 [40]	345	345	345		69	8.4	
Langenberg 2022 [41]	253	302	302		81.9	17.4	16
Parsons 2022 [42]	1056	1000	1000	5.3	31	13	
Summers 2022 [43]	126	127	127		65.1	30.6	
Ecker 2023 [44]	266	266	263	1.1	59	10	7
Tao 2023 [45]	142	142	133	5	31	10	9
Turco 2023 [46]	64	64	64		42	18.75	
Villani 2023 [47]	300	348	348		56	12.33	
Vodicska 2023 [48]	103	100	100		72	56	
Wadensten 2023 [49]	117	118	118		90	26.5	8.5
